# Real-time mental stress detection using multimodality expressions with a deep learning framework

**DOI:** 10.3389/fnins.2022.947168

**Published:** 2022-08-05

**Authors:** Jing Zhang, Hang Yin, Jiayu Zhang, Gang Yang, Jing Qin, Ling He

**Affiliations:** ^1^College of Biomedical Engineering, Sichuan University, Chengdu, China; ^2^Centre for Smart Health, School of Nursing, The Hong Kong Polytechnic University, Hong Kong, Hong Kong SAR, China

**Keywords:** stress detection, objective indicators, multimodality fusion, deep learning, matrix eigenvector

## Abstract

Mental stress is becoming increasingly widespread and gradually severe in modern society, threatening people’s physical and mental health. To avoid the adverse effects of stress on people, it is imperative to detect stress in time. Many studies have demonstrated the effectiveness of using objective indicators to detect stress. Over the past few years, a growing number of researchers have been trying to use deep learning technology to detect stress. However, these works usually use single-modality for stress detection and rarely combine stress-related information from multimodality. In this paper, a real-time deep learning framework is proposed to fuse ECG, voice, and facial expressions for acute stress detection. The framework extracts the stress-related information of the corresponding input through ResNet50 and I3D with the temporal attention module (TAM), where TAM can highlight the distinguishing temporal representation for facial expressions about stress. The matrix eigenvector-based approach is then used to fuse the multimodality information about stress. To validate the effectiveness of the framework, a well-established psychological experiment, the Montreal imaging stress task (MIST), was applied in this work. We collected multimodality data from 20 participants during MIST. The results demonstrate that the framework can combine stress-related information from multimodality to achieve 85.1% accuracy in distinguishing acute stress. It can serve as a tool for computer-aided stress detection.

## Introduction

Stress is an individual’s adaptation response to internal or external threats (Freeman, 1986; Mitra, 2008). It can affect people’s daily performance, memory, and decision-making abilities (Sharma and Gedeon, 2012; Nigam et al., 2021). Acute stress occurs when people are faced with urgent tasks such as mental arithmetic, academic exams, or public speaking (Allen et al., 2017). It usually disappears when the urgent task is over. If acute stress continues to permeate a person’s life, it can result in decreased physical and mental health and even lead to immune system disorders, cardiovascular disease, depression, or other diseases (Sauter et al., 1990; Segerstrom and Miller, 2004; van Praag, 2004; Heraclides et al., 2012; Beanland et al., 2013; Steptoe and Kivimäki, 2013). In modern society, stress has become increasingly widespread and severe. The European Union has established it as one of the most common causes of health problems (Wiegel et al., 2015).

To avoid harm to people caused by chronic or acute stress, it is essential to detect people’s stress state as early as possible and prevent the adverse effects of stress on people. Psychological evaluation of stress can be used to detect an individual’s stress state (Alberdi et al., 2016). Stress is assessed by filling out a questionnaire or talking to a psychologist. Since psychological evaluation is instantaneous and subjective, it often leads to false or even incorrect stress detection and is unable to meet the requirements of real-time detection (Ren et al., 2019). In contrast, using objective indicators such as physiological signals and behavioral information to detect stress is not affected by subjective influence (Setz et al., 2010; Cinaz et al., 2011; McDuff et al., 2012; Wei, 2013; Sharma et al., 2021).

When people are under stress, the autonomic nervous system (ANS) is stimulated and regulates involuntary body functions (Tsigos and Chrousos, 2002). As a result of changes in involuntary body functions, the electrocardiogram (ECG), voice, and facial expressions of people are affected. ECG is the physiological signal that can record cardiac activity. As regulated by the ANS, during stress, the heart rate increases, and the heartbeat’s standard deviation becomes larger (De Rosa, 2004; de Santos Sierra et al., 2011). These changes can be presented by ECG. Dominated by the ANS during stress, the pitch and speaking rate of voice are affected, while the energy and spectral characteristics of voice also change. The mean value, standard deviation, range of pitch increase, and jitter of pitch decrease when people are under stress. The spectral centroid goes up, and energy is concentrated in higher frequency bands (Lu et al., 2012). Likewise, affected by stress, facial expressions involving the eyes, mouth, and cheeks are different from calm (Liao et al., 2005; Sharma and Gedeon, 2012; Sundelin et al., 2013; Pampouchidou et al., 2016). The overall changes in these multiple facial regions constitute changes in facial expressions.

In recent years, multimodality fusion methods have received increasing attention. It has been widely used in computer-aided diagnosis and performs better prediction than single-modality-based methods (Yang et al., 2013; Vidya et al., 2015; Zhu et al., 2020). Since the ECG, voice, and facial expressions describe stress changes in a different way and are jointly affected by the ANS (Giannakakis et al., 2017). Fusing these multimodalities can detect the stress state from multiple aspects.

Deep learning technology has shown excellent performance in many fields (LeCun et al., 2015; Hatcher and Yu, 2018). Different from the handcrafted feature engineering methods, it automatically extracted the features of input through the deep learning network to minimize the feature extraction process and achieve better generalization ability. Due to the advantages of deep learning, a growing number of researchers are trying to use deep learning technology to detect stress (Jin et al., 2016; Hwang et al., 2018; Winata et al., 2018). Convolutional neural networks (CNNs) are an attractive way to distinguish different classes of inputs in deep learning technology. CNNs can extract features in multiple dimensions of the input, among which 2D-CNN can capture the global and local spatial information, and 3D-CNN can also capture the temporal information. Studies have proven that CNNs are effective for stress detection (Jin et al., 2016; Hwang et al., 2018; Winata et al., 2018), but the potential of using CNNs that fuse multimodality for stress detection remains to be explored.

In this work, a real-time deep learning framework that fused ECG, voice, and facial expressions for acute stress detection is proposed. Furthermore, we designed the temporal attention module (TAM) to find the keyframes related to stress detection in facial expressions. The proposed framework avoids complicated feature extraction and only requires simple preprocessing. The contributions of our work can be summarized as follows:

(1) This work proposes a deep learning framework that combines ECG, voice, and facial expressions for acute stress detection. The fusion method is based on the matrix eigenvector, which achieves 85.1% detection accuracy.

(2) The proposed framework utilizes TAM. The TAM assigns different learnable weights to different frames of facial expressions to highlight the distinguishing temporal representation for facial expressions about stress.

This research is organized as follows. The section “Materials and methods” introduces multimodality data acquisition, preprocessing, and the real-time deep learning framework. Section “Results” shows the results of our experiment, and Sections “Discussion” and “Conclusion” present the discussion and conclusion of our research, respectively.

## Materials and methods

### Materials

To collect multimodality data from people under stress, it is necessary to stimulate stress in participants by designed experiments (Setz et al., 2010; Tomova et al., 2016; Smets et al., 2018; Stepanovic et al., 2019). Many different stress-induced methods have been validated to stimulate stress. The most commonly used experimental paradigms are the Stroop Color-Word Interference Test and the Montreal Imaging Stress Task (MIST; Aitken, 1969; Lovallo, 1975; Kirschbaum et al., 1993; Renaud and Blondin, 1997; Dedovic et al., 2005; Reinhardt et al., 2012; Smeets et al., 2012; Tanosoto et al., 2012).

MIST is the gold standard experiment for stimulating stress. As a well-established psychological experiment employed in stress assessment, it has been proven to put people into a stress state by measuring the amount of cortisol in their saliva (Lederbogen et al., 2011; Kiem et al., 2013; Sioni and Chittaro, 2015). To date, a large number of studies on stress have been carried out on the basis of MIST and its modified experiments (Boehringer et al., 2015; Chung et al., 2016; Wheelock et al., 2016; Gossett et al., 2018; Hakimi and Setarehdan, 2018; Li et al., 2018; Xia et al., 2018; Noack et al., 2019; Perez-Valero et al., 2021). The MIST is a computer-based standardized psychological experimental designed to assess the effects of psychological stress on people’s physiology and behavior (Dedovic et al., 2005). To obtain multimodality data from participants in a stressful state, this work used MIST to induce people to be under stress.

#### Participants

Twenty right-handed participants (11 males, 9 females, mean age = 22.75, SEM = 0.13, age-range 20–25 years, 20 Chinese) participated in the MIST to stimulate psychological stress. All the participants were participating in MIST for the first time. The overall flow of MIST was introduced before the experiment started.

#### Data collection

MIST is a computer-based psychological experimental paradigm that mainly includes (1) the calm stage, (2) the control stage, (3) the experimental stage, and (4) the recovery stage.

**FIGURE 1:**
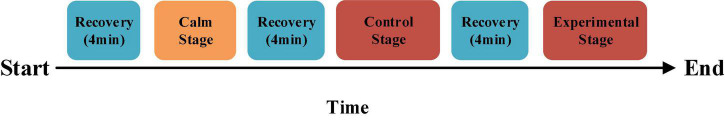
The Montreal imaging stress task process.

In the calm stage, the participants read the equation with the answer. In the control stage, the participant clicks on the correct answer in the program and reads out the calculation and the result, and the screen will display correct or incorrect. During the experimental stage, the participants perform calculations with time constraints, and the program adaptively adjusts the time constraints and difficulty. If the participants correctly solve three arithmetic tasks in a row, the program will reduce the time constraints and raise calculation difficulty. Both the control stage and the experimental stage can cause psychological stress in the participants. The recovery helps participants return to a calm state. After the MIST experiment, each participant was asked to fill out a stress questionnaire. Figure 1 shows the MIST process.

This work used the MIST program written and deployed using JDK 8u66 for Windows. The program can automatically create arithmetic tasks including addition, subtraction, multiplication, and division. There are five categories of difficulty for calculation problems, the two easiest of which are the addition or subtraction of 2 or 3 one-digit integers. The two classes of medium difficulty contain 3 or 4 integers and allow for multiplication. For the most difficult category, the calculation includes addition, subtraction, multiplication, and division of four integers. The answers to all computational tasks are integers between 0 and 9.

**FIGURE 2:**
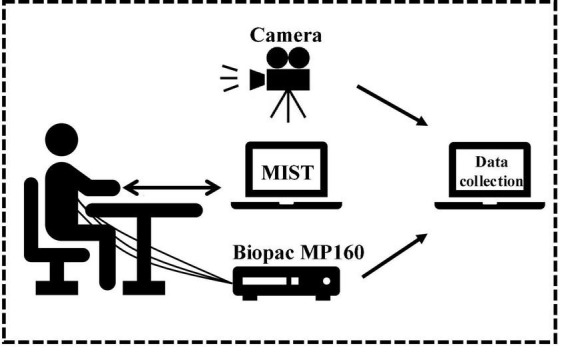
Data collection platform.

The multimodality data collection platform used in this work includes two computers (one for the MIST experiment and one for collecting data), a physiological signal acquisition device Biopac MP160, and a Sony video camera FDR-AX700. The MP160 contains the participant’s ECG signal through a wireless transmission module and transmits data to the collecting computer through the network cable. The ECG signal is acquired by three-electrode leads, two electrodes placed symmetrically in the fourth or fifth rib region, and one electrode placed in the right upper chest area, where the sampling frequency is 2000 HZ. The camera captures the facial expressions and voice of the participants during the MIST experiment and sends them to the collecting computer, where the resolution of the video is 1920*1080 and 30 fps. The camera and sensor are turned on at the same time, aligning the data according to the data and video length. The platform diagram is shown in Figure 2.

The process of participants completing one equation is considered as a piece of data. A piece of the sample contains participants’ facial expressions, ECG, and voice. The samples were labeled according to the stage of MIST. In this way, 1271 samples were acquired, including 531 labeled “calm” and 740 labeled “stress.”

### Methods

**FIGURE 3:**
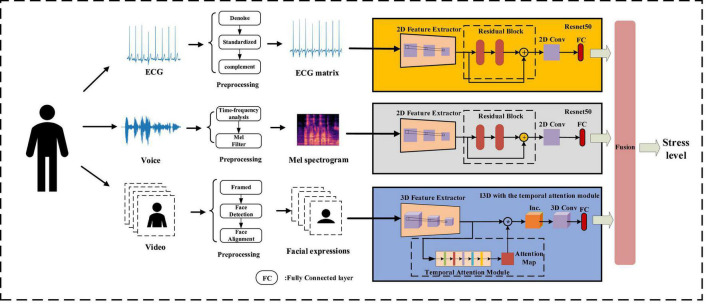
Multimodality stress detection.

The real-time deep learning framework proposed in this work used ECG, voice, and facial expressions for stress detection. Each modal was preprocessed before being input into the framework. ECG and voice were converted into the form that represents their time-frequency changes, and facial expressions were extracted from the collected video. Then the multidimensional features of each modality were extracted through the deep learning framework. The fully connected layers in the framework obtained the information about the stress state, and the framework fused them into a global matrix for stress detection. An overview of multimodality stress detection in this work is shown in Figure 3.

#### Data preprocessing

##### Facial expressions preprocessing

This work removed background information to isolate the facial area, which can avoid being disturbed by irrelevant information of surrounding noise and clutter in different scenarios of reality. Video **V_i_** was framed into sequence of image frames**V_i_**=(**frame**_**i1**_, **frame**_**i1**_,…,**frame**_**in**_), and the face area was detected by MTCNN ^[45]^ on each image frame and then aligned. **Face_i_**=(**face**_**i1**_,**face**_**i1**_,…,**face**_**in**_) denotes a sequence of face images detected from sequence **V_i_**.

##### Electrocardiogram preprocessing

**FIGURE 4:**
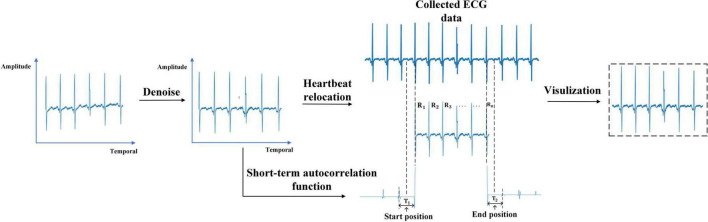
Electrocardiogram preprocessing.

The original ECG signal contains high-frequency and electrical noise. And intercepting the ECG signal according to temporal leads to the start position and the end position of the heartbeat in different samples being inconsistent. We preprocessed the original ECG signal below to solve these problems. The preprocess of ECG is shown in Figure 4.

(1) Denoising: The ECG signal was passed through a notch filter with a cutoff frequency of 50 Hz to eliminate the interference caused by the industrial frequency current. The energy of the ECG signal is concentrated in the frequency band of less than 50Hz. Filtering out the ECG signal higher than 50Hz will not affect the expression of ECG changes. Then a pass filter with a cutoff frequency of (0.5, 50) Hz was used to reduce the influence of electrode noise, muscle noise, and baseline wander noise on the ECG signal. After that, the ECG signal was standardized to eliminate amplitude scaling in the heartbeat cycles.

(2) Heartbeat relocating: Due to the interception according to the temporal causes, the start position and the end position of the heartbeat in different samples were inconsistent. In this work, the start position and end position of the ECG signal were relocated based on Niu’s work (Niu et al., 2020). It takes the middle temporal position between the first **R** wave in the sample and its preceding **R** wave in the collected ECG data as the start position. The middle temporal position between the last **R** wave in the sample and the **R** wave after it in the collected ECG data is considered the end position. The short-term autocorrelation function is used to calculate the temporal distance between two **R** waves (Piotrowski and Różanowski, 2010).

First, We detected the temporal position of each **R** wave in the ECG signal through a sliding window with a threshold. The temporal position of the **R** waves was recorded as**R_1_**,…,**R_N_**. Second, we reversed the ECG signal 2 s before the **R_1_** and calculate the short-term autocorrelation function as **X_1_**(**n**). The short-term autocorrelation function of the ECG signal 2 s after the **R_5_** was calculated as **X_N_**(**n**). **X_1_**(**n**) and **X_N_**(**n**) were clipped using thresholds α=**0.1** and β=−**0.1** with the following formula:


$$\text{X}\,\left(n\right)=\left\{ \begin{array}{c} \text{x}\,\left(n\right)-\alpha ,\text{x}\,\left(n\right)>\alpha \\ 0,\beta \leq \text{x}\,\left(n\right)\leq \alpha \\ \text{x}\,\left(n\right)-\beta ,\text{x}\,\left(n\right)<\beta \end{array}\right.$$

The temporal between the first sample and the maximum sample of the first harmonic in **X_1_**(**n**) represents the temporal distance **T_1_** between **R_1_** and its preceding **R** wave. We take the middle temporal position of **T_1_** as the start position. The temporal between the first sample and the maximum sample of the first harmonic in **X_N_**(**n**) represents the temporal distance **T_2_** between **R_n_** and the **R** wave after it. The middle temporal position of T2 was taken as the end position of the heartbeat.

(3) Visualization: The relocated heartbeat were converted into the form of an image. The vertical axis represents the amplitude of the heartbeat and the horizontal axis represents the temporal of the heartbeat.

##### Voice preprocessing

The Mel spectrogram calculated in the time-frequency domain analysis contains time and frequency information of voice. It converts the linear frequency scale into a logarithmic scale and represents the distribution of signal energy on the Mel-scale frequency, which is similar to human hearing. Mel spectrogram can intuitively show the spectral changes of voice over time. Therefore, we convert the voice into the Mel spectrogram.

After the voice data are divided into each sample, we pre-emphasis the data. Then, the Hamming window of 30ms length is used to frame the data with 15 ms overlap.

After that, we calculate energy density using the short-time Fourier transform (STFT) and transform the frequency into Mel-scale band to extract Mel spectrogram.

#### Real-time deep learning framework for stress detection

The real-time deep learning framework was developed by combining ResNet50 (He et al., 2016) and I3D with the temporal attention module. ResNet50 extracts the global and local features of the ECG matrix and Mel spectrogram through identity mapping. I3D with the temporal attention module learns the spatiotemporal changes in facial expressions, and the temporal attention module enables I3D to extract important temporal features.

The stress state information in their fully connected layers was combined into a global matrix, leading to a multimodal stress information representation. The multimodality information about stress was fused for stress detection based on matrix eigenvectors.

#### ResNet50

**FIGURE 5:**
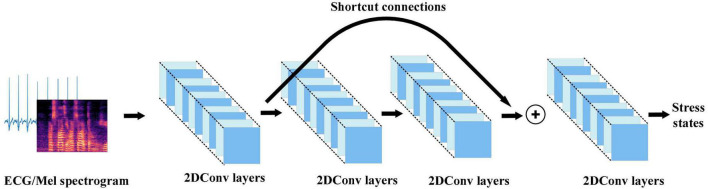
2D convolution strategy for electrocardiogram and Mel spectrogram input in this work.

Converting the ECG and voice signals into three-dimensional matrices can represent higher-order and nonlinear characteristics of the signals. This work used Resnet in 2D-CNN to extract multiple features from the ECG and Mel spectrogram. ResNet avoids the problem of gradient disappearance and explosion in traditional 2D-CNN through shortcut connections. The 2D convolution strategy of ECG and Mel spectrogram input in this work is shown in Figure 5.

The 2D-CNN has a convolution layer composed of 2D convolution kernels. The convolutional layer can extract features in multiple dimensions to obtain the feature representation of the internal structure of the ECG and Mel spectrogram by scanning them with the 2D convolution kernels and reducing the number of parameters through local connectivity and parameter sharing. The 2D convolution can be expressed as:


$${\text{v}}_{\mathrm{ij}}^{\mathrm{xy}}=\text{f}\,\left({\text{b}}_{\mathrm{ij}}+\sum_{m}\sum_{p=0}^{P_{i}-1}\sum_{q=0}^{Q_{i}-1}{\text{W}}_{\mathrm{ijm}}^{\mathrm{pq}}\,{\text{v}}_{\left(i-1\right)\,m}^{\left(x+p\right)\,\left(y+q\right)}\right)$$

where ${\text{v}}_{\mathrm{ij}}^{\mathrm{xy}}$ is the **i** convolution result at the **j**position in feature map (**x**,**y**) of the layer; **f** is the activation function Rectified Linear Unit (ReLU; Nair, 2010); **b_ij_** is the deviation of the feature map; **m** is the index of the feature map in layer **i**−**1**; **Q_i_**, **P_i_** is the height and width of the convolution kernel; and ${\text{W}}_{\mathrm{ijm}}^{\mathrm{pq}}$ represents the value at the position of the feature map.

In traditional 2D-CNN, when the network structure becomes very deep, there will be unavoidable problems of gradient disappearance or explosion, and the problem of accuracy saturation or decline. This causes such networks to be unable to capture the overall stress information in the input. Resnet avoids the problems caused by a network structure that is too deep through the residual block with shortcut connections. The residual block connects the inputs in the lower layers and high layers which converts the input maps into identity maps.

**FIGURE 6:**
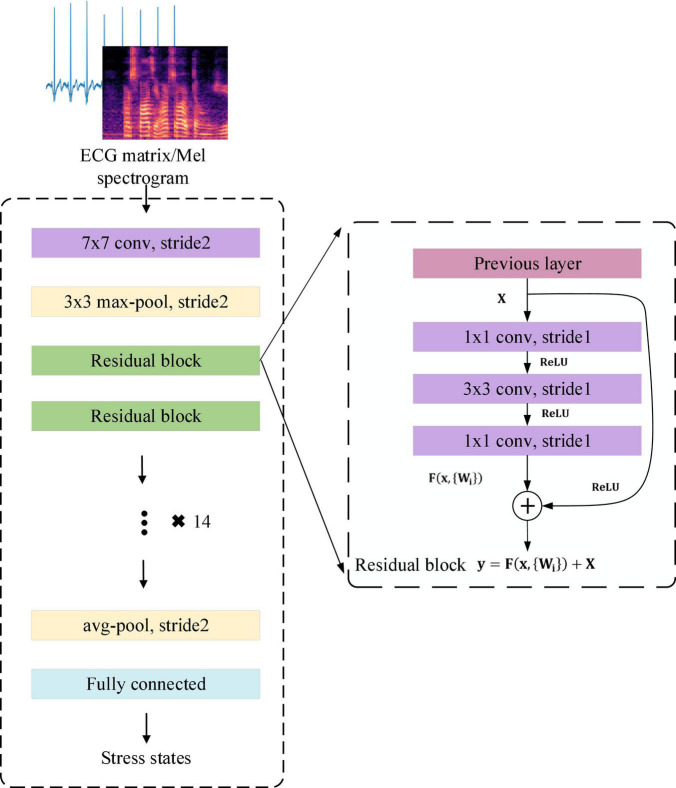
The overall architecture of the ResNet50.

This work used ResNet50 to extract effective representations of stress in ECG and Mel spectrogram input. ResNet50 has half the floating-point operations (FLOPs) of ResNet101, and only 5% more than ResNet34. It reduces the number of FLOPs while satisfying the accuracy requirements. The overall architecture of ResNet50 is shown in Figure 6.

The residual block in Figure 6 is defined as:


$$\text{y}=\text{F}\,\left(x,\left\{{\text{W}}_{i}\right\}\right)+\text{X}$$

where **X** is the input of weight layer;**ReLU**is the activation function; **F**(**X**,{**W_i_**}) is the output after three convolution layers. Identity mapping adds **F**(**X**) and **X** as the input **y** to the next residual block.

### I3D With the Temporal Attention Module

**FIGURE 7:**
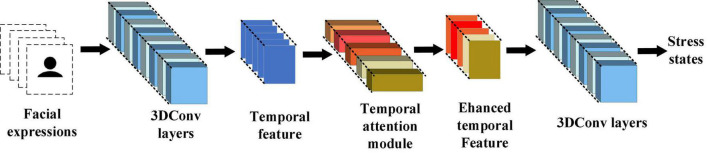
3D convolution strategy for facial expressions input in this work.

As mentioned above, detecting stress by facial expressions requires comparing temporal changes in multiple facial regions, including the eyes, mouth, and cheeks. This work used the inflated 3D-CNN (I3D) with the temporal attention module to learn the overall changes in facial expressions during stress. The I3D extracts the features of stress in facial expressions and the temporal attention module tells I3D which frames are important. The 3D convolution strategy for facial expressions input in this work is shown in Figure 7.

The 3D-CNN has a 3D convolution kernel that can analyze successive frames of facial expressions and capture spatiotemporal features of facial expressions. The 3D convolution can be expressed as:


$${\text{v}}_{\mathrm{km}}^{\mathrm{xyz}}=\text{f}\,\left({\text{b}}_{\mathrm{km}}+\sum_{p=0}^{P_{k}-1}\sum_{q=0}^{Q_{k}-1}\sum_{r=0}^{R_{k}-1}{\text{w}}_{\mathrm{kmn}}^{\mathrm{pqr}}\,{\text{u}}_{\left(k-1\right)\,n}^{\left(x+p\right)\,\left(y+q\right)\,\left(z+r\right)}\right)$$

where ${\text{v}}_{\mathrm{km}}^{\mathrm{xyz}}$ is the position **k** in the **m** feature map of the (**x**,**y**,**z**) layer; **f** is the loss function; **u** is the input from the **k**−**1** to **k** layer; **P_k_**,**Q_k_**,**R_k_** are the width height and depth of the convolution kernel size; **b_km_** is the deviation of the ${\text{w}}_{\mathrm{kmn}}^{\mathrm{pqr}}$ feature map.

In traditional 3D-CNN (C3D), each layer generally uses a single-size convolution kernel and forward propagation, which cannot extract overall features. This results in the entire facial expression being ineffectively represented. The inflated 3D-CNN (I3D; Carreira and Zisserman, 2017) combines the advantages of GoogLenet (Szegedy et al., 2015) and 3D-CNN. It uses the inflated inception module for feature extraction. The inflated inception module uses convolution kernels of different sizes to extract features, and finally concatenates them to increase the network’s ability to extract overall features. Therefore, for facial expressions, the corresponding features of stress in multiple facial regions can be extracted by I3D.

During facial expression changes, subtle changes tend to last only a few frames, so not all frames are equally important for distinguishing facial expressions. The identical scale is used to pool temporal information in traditional I3D, which makes important frames lost and trapped in local details.

**FIGURE 8:**
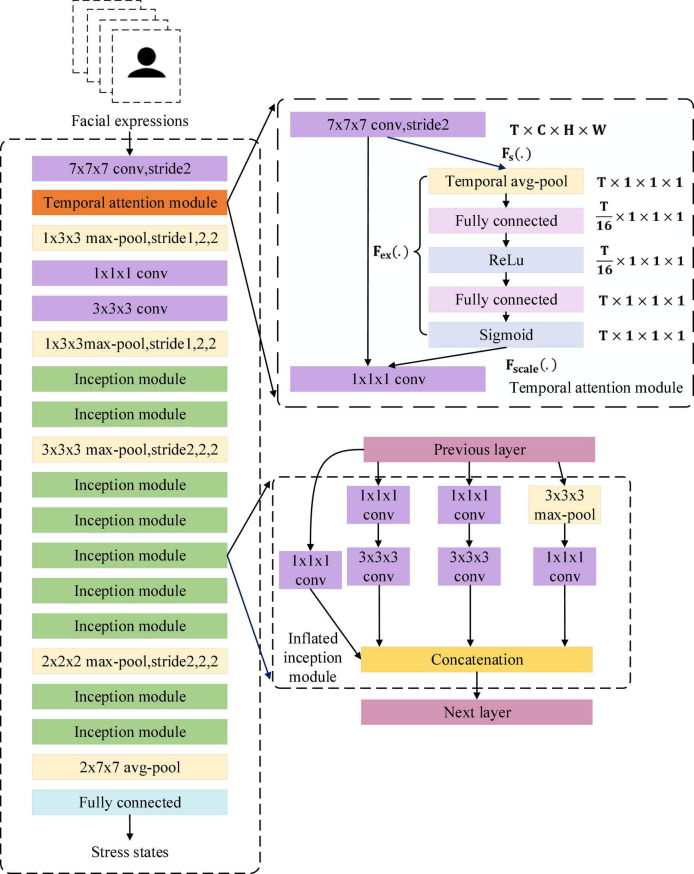
The overall architecture of the I3D with temporal attention module.

To enhance the global perception of temporal information in I3D and avoid getting caught in local temporal details, this work proposed the temporal attention module (TAM) for the I3D layers. In TAM, global pooling is calculated for each input frame. Then two fully connected layers and a sigmoid function are used to generate a temporal attention map, which is finally combined with the multiplication of the original feature maps to change the proportion of temporal information captured by the initial layer of I3D. It makes I3D highlight the distinguishing features while ignoring interfering features when extracting the temporal information of facial expressions. The overall architecture of I3D with TAM is shown in Figure 8.

Formally, a statistic **z** ∈ **R^T^** is generated by shrinking **u** ∈ **R^C×H×W^** (**F_s_**(.)**)** through its channel and spatial information **C**×**H**×**W**:


$${\text{z}}_{t}={\text{F}}_{s}\,\left({\text{u}}_{t}\right)=\frac{1}{\text{C}\times \text{H}\times \text{W}}\,\sum_{i=1}^{C}\sum_{j=1}^{H}\sum_{k=1}^{W}{\text{u}}_{t}\,\left(i,j,k\right)$$

To use the shrinking information, we follow it with a second operation **F_ex_**(.). We want to ensure that different frames are allowed to be emphasized, so we choose sigmoid activation as a simple gating mechanism:


$$\text{S}={\text{F}}_{\mathrm{ex}}\,\left(z,W\right)=\sigma \,\left(\text{g}\,\left(z,W\right)\right)=\sigma \,\left({\text{W}}_{2}\,\delta \,\left({\text{W}}_{1}\,\text{z}\right)\right)$$

where δrefers to the ReLU function. ${\text{W}}_{1}\in {\text{R}}^{\frac{T}{r}\times T}$ and ${\text{W}}_{2}\in {\text{R}}^{T\times \frac{T}{r}}$, where **r** is the reduction ratio. The final output of the TAM is acquired by multiplying **S** and **u**:


$$\text{Output}={\text{F}}_{\mathrm{scale}}\,\left({\text{u}}_{t},{\text{S}}_{t}\right)={\text{S}}_{t}\,{\text{u}}_{t}$$

### Multimodality fusion

This work proposed a fusion method based on the eigenvector corresponding to the largest eigenvalue of the matrix. In our method, the posterior probability information of each stage for each modality is composed as a vector. The vectors of the three modalities are composed into the stress information matrix. Our method uses the normalized eigenvector corresponding to the largest eigenvalue of the matrix as the weight to fuse the multimodality information.

After Resnet50 and I3D with TAM extracted input features about stress, their fully connected layer can obtain the posterior probability information of the stress stage of the input. For each modality, the probability of each stage **P_calm_**,**P_control_**,**P_experimental_** derived from this modality can be composed as a vector, and the vectors of the three modalities **V_voice_**,**V_face_**,**V_ECG_** can be composed as a matrix, which contains probabilistic information about each stage of each modal. We define this matrix as the stress information matrix **M_global_**.


$${\text{M}}_{\text{global}}=({\text{V}}_{\text{Voice}},{\text{V}}_{\text{Face}},{\text{V}}_{\text{ECG}})$$

$$=\left(\begin{array}{ccc} {\text{P}}_{\text{calm}\_\text{V}}\text{,} & {\text{P}}_{\text{calm}\_\text{F}}\text{,} & {\text{P}}_{\text{calm}\_\text{E}}\\ {\text{P}}_{\text{control}\_\text{V}}, & {\text{P}}_{\text{control}\_\text{F}}, & {\text{P}}_{\text{control}\_\text{E}}\\ {\text{P}}_{\text{experimental\_V}}, & {\text{P}}_{\text{experimental\_F}}, & {\text{P}}_{\text{experimental\_E}} \end{array}\right)$$

In **M_global_**, the eigenvalues of **M_global_** indicate how much the probability is scaled, and the eigenvectors of **M_global_** indicate the direction of the probability. Compared with other eigenvalues and their corresponding eigenvectors, the largest eigenvalue and its corresponding eigenvector indicate that the probability in this direction is amplified to the greatest extent, that is, the probability of this matrix in this direction is the largest. Given this property of them, we use the eigenvector corresponding to the largest eigenvalue in **M_global_** to calculate the weight vector to fuse the multimodality information from **M_global_** for stress detection.

In **M_global_**, the probability of the three stages of MIST is included. The eigenvector **W_max_** represents the eigenvector corresponding to the largest eigenvalue in **M_global_**. We normalize **W_max_** as the weight vector **W_weight_**. **w_calm_**,**w_control_**,**w_experimental_** represent the weight values of the calm, control, and experimental stages, respectively.


$${\text{M}}_{\mathrm{global}}\to {\text{W}}_{\max}\overset{\mathrm{normalize}}{⟶}{\text{W}}_{\mathrm{weight}}=\left({\text{w}}_{\mathrm{calm}},{\text{w}}_{\mathrm{control}},{\text{w}}_{\mathrm{experimental}}\right)$$

After obtaining the weight values, the weight values are used to construct a diagonal weight matrix **W**.


$$\text{W}=\left(\begin{array}{c} {\text{w}}_{\mathrm{calm}}\\ 0\\ 0 \end{array}\,\begin{array}{c} 0\\ {\text{w}}_{\mathrm{control}}\\ 0 \end{array}\,\begin{array}{c} 0\\ 0\\ {\text{w}}_{\mathrm{experimental}} \end{array}\right)$$

The cross-modal global matrix **M_global_** is multiplied with the weight matrix to obtain the weighted matrix **W_global_**.


$${\text{W}}_{\mathrm{global}}=\text{W}*{\text{M}}_{\mathrm{global}}=\left({\text{w}}_{\mathrm{calm}},{\text{w}}_{\mathrm{control}},{\text{w}}_{\mathrm{experimental}}\right)$$

$$\left({\text{w}}_{\text{calm}},{\text{w}}_{\text{control}},{\text{w}}_{\text{experimental}}\right)$$

$$=\left(\begin{array}{ccc} {\text{W}}_{\text{calm}\_\text{V}}\text{,} & {\text{W}}_{\text{calm}\_\text{F}}\text{,} & {\text{W}}_{\text{calm}\_\text{E}}\\ {\text{W}}_{\text{control}\_\text{V}}, & {\text{W}}_{\text{control}\_\text{F}}, & {\text{W}}_{\text{control}\_\text{E}}\\ {\text{W}}_{\text{experimental\_V}}, & {\text{W}}_{\text{experimental\_F}}, & {\text{W}}_{\text{experimental\_E}} \end{array}\right)$$

**FIGURE 9:**
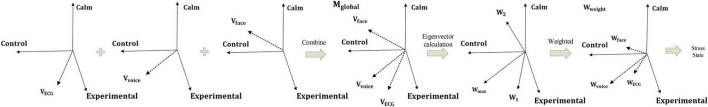
The fusion method based on matrix eigenvector.


In **W_global_**, **w_calm_**=(**w**_**calm**−**V**_,**w**_**calm**−**F**_,**w**_**calm**−**E**_) represents the weighted posterior probability of the calm stage. **w_control_**=(**w**_**control**−**V**_,**w**_**control**−**F**_,**w**_**control**−**E**_) represents the weighted posterior probability of the control stage. **w_experimental_**=(**w**_**experimental**−**V**_,**w**_**experimental**−**F**_,**w**_**experimental**−**E**_) represents the weighted posterior probability of the experimental stage. Whether the value of **W_global_**(**w_i_**) is positive or negative, it’s in the direction of the represented stage axis, the absolute size of its value represents the probability in this stage. Our method’s output is the stress state corresponding to the absolute maximum in **W_global_**. The fusion process is shown in Figure 9.

$$\text{Output=max}\left[\left|\sum_{i=1}^{3}{\text{w}}_{\text{calmi\_i}}\text{,}\sum_{i=1}^{3}{\text{w}}_{\text{controli\_i}}\text{,}\sum_{i=1}^{3}{\text{w}}_{\text{experimental\_i}}\right|\right]$$

## Results

The performance of the proposed deep learning framework for stress detection was evaluated with the collected dataset through four sets of experiments, including multimodality stress detection, single-modality-based stress detection, the effectiveness of the temporal attention module in I3D, and comparison with different models. The 10-fold-cross-validation method was utilized on the dataset for cross-validation to ensure the generalization ability of our method.

### Implementation details

This work randomly divided 80% of the data after leaving one-fold out into the training set and 20% into the validation set. The experimental environment is 64-bit Windows 10, GeForce RTX2070, and 16 GB memory. Our implementation is based on Python version 3.6.13 and PyTorch version 1.9.0 with CUDA version 11.4.

### ResNet50 parameter settings

During training, the trainable parameters in ResNet50 were initialized with the uniform random distribution. For the ECG matrix and Mel spectrogram, the input size is 307 × 230. They are randomly cropped at 224 × 224 and flipped horizontally for better training. The network was trained by Adam optimization. The learning rate is 0.0001. The network is trained using a batch size of 32 for 80 epochs.

During validation, the input of the ECG matrix and Mel spectrogram are also center-cropped at the same size of training without horizontal flipping.

### I3D with temporal attention module parameters settings

During training, the trainable parameters in I3D with the temporal attention module were also initialized with a uniform random distribution. For facial expressions, 64 consecutive frames of facial expressions are randomly sampled from each video. Input frames are rescaled to 224 * 270 and randomly cropped to 224 * 224. Frames are randomly flipped horizontally for data augmentation to improve the invariance properties of geometric perturbations. The network was trained by the Adam optimization. The learning rate is 0.01. The network is trained using batch size 1*3*64 for 30 epochs.

During validation, the input frames of facial expressions are sampled at the fixed central location of each video. These frames are rescaled and center-cropped at the same size of training without horizontal flipping.

### Multimodality stress detection

The deep learning framework fused ECG, voice, and facial expressions for stress detection. Information about stress in multimodality can be obtained from the fully connected layer of ResNet50 and I3D with the temporal attention module in the framework. The framework integrated them into a global matrix for representation and used the fusion method based on matrix eigenvectors to detect stress.

The performance of the proposed deep learning framework and every single-modality-based method in the framework for stress detection was compared in the terms of four widely used metrics: accuracy, precision, recall, and F1-score. As expected, the multimodality method provided the best performance in stress detection, suggesting that the deep learning framework using multimodality data for stress detection can achieve better stress detection performance than the single-modality-based method.

As illustrated in Table 1, stress detection using multimodality improved performance compared to using only single-modality data. The accuracy of multimodality result is increased from the highest accuracy of the single-modality result of 83.9–85.1%. Revealing the deep learning framework can efficiently fuse the multimodality information for stress detection and is more effective than single-modality-based methods.

**TABLE 1:** Stress detection accuracy, precision, recall and F1-score using single- and multimodality data.

Modal	Accuracy	Precision	Recall	F1-Score
ECG	0.741	0.737	0.743	0.731
Voice	0.830	0.825	0.829	0.827
Facial expressions	0.792	0.795	0.803	0.799
**Fusion**	**0.851**	**0.857**	**0.866**	**0.861**

The bold values are the result of the multimodality stress detection.

Moreover, the results demonstrated that using either single-modality or multimodality all can effectively detect stress. This work can use either of them to distinguish between calm and stress, which allows the method to be applied in situations where multimodality data are not available.

### Single-modality stress detection

Stress causes changes in people’s involuntary body functions, and each modal of the changes can be used for stress detection. Compared with the handcrafted feature engineering methods, the deep learning network can automatically extract multiple features of the input. This work explored the use of Resnet50 and I3D with the temporal attention module to extract features in ECG, voice and facial expressions for stress detection.

After feature extraction, each single-modality was used for stress detection after simple preprocessing. The confusion matrices of each single-modal based method are shown in Table 2, and the matrices are also compared with the multimodality-based method.

**TABLE 2:** Stress detection confusion matrix of the single-modal and multimodality methods.

Actual labels	Predicted labels							
	ECG		Voice		Facial expressions		Fusion	
	Calm	Stress	Calm	Stress	Calm	Stress	Calm	Stress
Calm	0.751	0.266	0.821	0.164	0.868	0.262	0.913	0.193
Stress	0.249	0.734	0.179	0.836	0.132	0.738	0.087	0.807

In the single-modality-based method, the best recognition of the calm state is achieved by facial expressions of 86.8%, and the best recognition of the stress state is achieved by voice of 83.6%. Furthermore, the matrices also prove that ResNet50 and I3D with TAM can effectively extract stress-related features in each modality after simple preprocessing.

### Effectiveness of the temporal attention module

To explore the effectiveness of TAM, an ablation experiment was designed that removed TAM for I3D with TAM. The confusion matrices of I3D without TAM are shown in Table 3. Compared with the confusion matrices of I3D with TAM in Table 2, the overall detection performance and the recognition of the calm state is improved with TAM, which proves that TAM can enhance the perception of temporal information between the calm state and stress state in I3D and emphasize the distinguishing temporal features in facial expressions.

**TABLE 3:** Stress detection confusion matrix of I3D without temporal attention module.

Actual labels	Predicted labels	
	Calm	Stress
Calm	0.824	0.261
Stress	0.176	0.739

**FIGURE 10:**
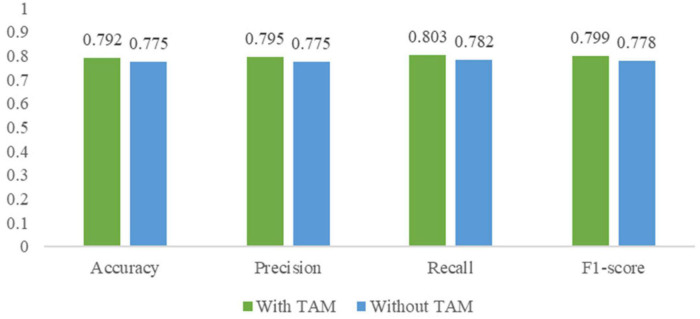
Performance comparison of I3D without temporal attention module (TAM) and I3D with TAM.

This work also compared the performance of I3D without TAM and I3D with TAM in terms of the above four widely used metrics. As shown in Figure 10, without TAM, the accuracy, precision, recall, and F1-score of stress detection by facial expressions dropped by approximately 1.7, 2.0, 2.1 and 2.1%, respectively. The result demonstrates the feasibility of the TAM in finding the more influential association between frames in facial expressions about stress and I3D with TAM can achieve better performance in stress detection.

### Time assessment for real-time applications

**FIGURE 11:**
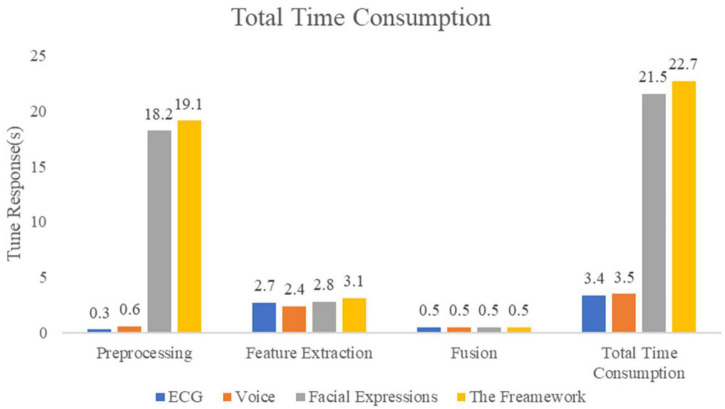
Time assessment for real-time applications.

This work also analyzed the time duration of the real-time deep learning framework to verify that real-time performance requirements are met. The results show that the framework meets the needs of real-time stress assessment. Each process present in the framework was evaluated which mainly consists of three parts, namely preprocessing, feature extraction, and multimodality fusion. The results of the time duration are visualized in Figure 11.

### Comparison with widely used convolutional neural networks

In this part, the comparison experiment is conducted with several widely used CNNs in each modality to evaluate the effectiveness of our work. The CNNs are ResNet101, GoogLeNet, EfficientNet, and C3D (Szegedy et al., 2015; Tran et al., 2015; He et al., 2016; Tan and Le, 2019), which are widely used and have been proven to have a strong performance. Table 4 presents their stress detection results.

**TABLE 4:** Stress detection accuracy, precision, recall and F1-score of several widely used convolutional neural networks.

Modal	Model	Accuracy	Precision	Recall	F1-score
ECG	ResNet101^[98]^	0.706	0.712	0.717	0.714
	GoogLeNet^[97]^	0.723	0.735	0.739	0.737
	EfficientNet^[99]^	0.769	0.773	0.781	0.777
	ResNet50^[98]^	0.741	0.737	0.743	0.740
Voice	ResNet101^[98]^	0.802	0.797	0.801	0.799
	GoogLeNet^[97]^	0.763	0.756	0.752	0.754
	EfficientNet^[99]^	0.809	0.804	0.812	0.808
	ResNet50^[98]^	0.830	0.825	0.829	0.827
Facial expressions	C3D^[100]^	0.582	0.582	0.500	0.538
	I3D^[101]^	0.775	0.775	0.782	0.778
	I3D with TAM	0.792	0.795	0.803	0.799

As is indicated in Table 4, the accuracy of ResNet101 for stress detection by ECG and voice are 70.6 and 80.2%, both of which are lower than ResNet50. This proves that although Resnet has shortcut connections, blindly increasing the network depth cannot greatly improve the performance and will also increase the number of FLOPs. GoogLeNet and EfficientNet achieved 72.3 and 76.3%, and 76.9 and 80.9% accuracy for stress detection using ECG and voice, respectively, owing to different structures being used to solve the problems of gradient disappearance or explosion.

Table 4 also shows that I3D with TAM outperforms I3D and C3D, achieving the highest accuracy for stress detection using facial expressions. Compared with C3D, I3D can better extract the overall features of facial expressions through the inflated inception module, while TAM can highlight the distinguishing information in the overall features and find the keyframes in facial expressions to achieve optimal stress detection performance. The I3D with TAM proposed in this work can simultaneously extract the distinguishing and overall features of facial expressions through the inflated inception module and TAM. The feature maps extracted by I3D with TAM have more information, which can better use facial expressions for stress detection.

Since ResNet50 achieve 83.0% accuracy for stress detection using voice and EfficientNet achieve 76.9% accuracy for stress detection using voice. We explore EfficienNet for stress detection using ECG and voice, and I3D with TAM for stress detection using facial expressions. The same fusion method is used to fuse multimodality information for stress detection. The results are shown in Table 5.

**TABLE 5:** Stress detection accuracy, precision, recall and F1-score by multimodality using different convolutional neural networks.

Fusion	Accuracy	Precision	Recall	F1-score
EfficientNetI3D with TAM	0.839	0.850	0.858	0.854
ResNet50 I3D with TAM	0.851	0.857	0.866	0.861

Although EfficientNet produces good performance in stress detection using ECG and voice, the comparison of the fusion results shown in Table 5 reveals that ResNet50-based fusion method can achieve better fusion accuracy. We prove that our proposed real-time deep learning framework based on ResNet50 achieves better performance in stress detection.

## Discussion

The automatic stress detection in people with objective indicators demonstrated that it can be reliable and does not require many human resources. It avoids the inference of stress detection caused by the instantaneous and subjective psychological evaluation. However, many stress detection research uses a single-modality-based for stress detection. For the multimodality data of people under acute stress, single-modality-based stress detection does not fully use all the collected data. Therefore, this work explored a way to fuse multimodality for acute stress detection.

In this work, a real-time deep learning framework was proposed to fuse multimodality for acute stress detection. Our work is trying to detect acute stress. We do not detect different levels of stress. This work proposed a fusion method based on matrix eigenvectors to fuse multimodality information. Furthermore, we designed the temporal attention module (TAM) to find the keyframes related to acute stress in facial expressions.

MIST can stimulate acute stress in both the control and experimental stage by measuring changes in participants’ cortisol. To evaluate the performance of the proposed framework, the multimodality dataset was collected from 20 participants during the MIST experiment. Compared to the number of participants in other stress detection research, many research collects stress data from 10-30 participants. Xia analyzed variations in both electroencephalogram (EEG) and ECG signals from 22 male subjects (Xia et al., 2018). Minguillon collected multiple biosignals from 10 subjects (Minguillon et al., 2018). Perez-Valero conducted a group of 23 participants over the MIST experiment (Perez-Valero et al., 2021). To confirm whether the participants developed acute stress during the MIST experiment, we asked each participant to fill out a questionnaire after the MIST experiment. In the questionnaires, all participants reported experiencing acute stress in both the control and experimental stages of MIST. Given the inter-individual differences in the reaction to MIST, we considered the acute stress generated in both phases as the same category. The data were labeled as “calm” and “stress” instead of different stress levels.

This work provides a method for stress detection using multimodality. The method achieves 74.1, 79.2, and 83.0% detection accuracy using ECG, facial expressions, and voice, respectively. In the single-modality-based method using facial expressions, using I3D with TAM achieves a 1.7% higher detection accuracy than using I3D. After the probability information in every single modality is fused by the proposed multimodality fusion method, the detection accuracy of 85.1% can be achieved. The results show that the overall stress detection performance is improved by using multimodality. In our results, the recognition of the stress state for the fusion method is lower than the single-modality-based method using voice. This is caused by the accuracy of the single-modality method using ECG and the accuracy of the single-modality method using voice. In our multimodality fusion method, the stress information matrix is constructed for each multimodality sample. For the recognition of the stress state, the single-modality method using ECG has an accuracy of 73.4%, and the single modality method using facial expressions has an accuracy of 73.8%. This leads to an increased probability that two or three modalities’ stress information in a sample is simultaneously opposed to the real state. When the main probability information of the two modalities is opposite to the real state, it will dominate the main probability direction of the stress information matrix. This caused the stress information matrix to be more likely at the opposite of the real state, leading to incorrect recognition results. The results in this work show that the overall stress detection performance is improved by using multimodality.

Compared with other methods using other signals, a variety of objective indicators are used for stress detection. The other signals such as EEG, electromyogram (EMG), and functional near-infrared spectroscopy (fNIRS) require special equipment and a lot of pre-collection preparations and post-collection work, it limits the practical application of these signals and is unpleasant for the experimental participants. Our work demonstrates the reliability of detecting acute stress using ECG, voice, and facial expressions. The results show that using those feasible multimodality can achieve 85.1% stress detection accuracy. In addition, the modalities used in this work are easy to be acquired in daily life.

## Conclusion

In this work, a real-time deep learning framework was proposed to fuse ECG, voice, and facial expressions for stress detection. The result shows that the fusion of multimodality information about stress can achieve 85.1% detection accuracy, which provides a reference for the research of multimodality stress detection based on deep learning technology in the future. The framework extracted the stress-related features of each modal through ResNet50 and I3D with TAM and gave different weights for each type of stress state according to the global stress information matrix. At the same time, this work designed the temporal attention module to find the more influential association between frames in facial expressions for stress detection. Compared with the optimal single-modality-based method, the accuracy of the multimodality result is improved by 2.1%. This work provides an objective reference for fusing multimodality to detect stress based on deep learning technology, and preventing stress from harming people’s physical and mental health.

## Data availability statement

The datasets presented in this article are not readily available because they contain identifiable information such as recognizable faces and must be approved by the Ethics Committee on Biomedical Research, West China Hospital of Sichuan University. Requests to access the datasets should be directed to LH, ling.he@scu.edu.cn.

## Ethics statement

The studies involving human participants were reviewed and approved by the Ethics Committee on Biomedical Research, West China Hospital of Sichuan University. The patients/participants provided their written informed consent to participate in this study. Written informed consent was not obtained from the individual(s) for the publication of any potentially identifiable images or data included in this article.

## Author contributions

All authors listed have made a substantial, direct, and intellectual contribution to the work, and approved it for publication.
